# A cross-sectional study of the self-report of stress among emergency department patients

**DOI:** 10.4103/0974-2700.55325

**Published:** 2009

**Authors:** Ted D Nirenberg, Janette Baird, Magdalena Harrington, Michael J Mello, Robert Woolard, Richard Longabaugh

**Affiliations:** 1 Department of Emergency Medicine, Warren Alpert Medical School of Brown University, USA; 2 Department of Psychiatry and Human Behavior, Warren Alpert Medical School of Brown University, USA; 3 Injury Prevention Center, Rhode Island Hospital, USA; 4 Texas Tech University Health Sciences Center, USA

**Keywords:** Stress, psychological, emergency service, hospital, adult, patients

## Abstract

**Background:**

There is a lack of information about the prevalence of stress and types of stressors experienced by Emergency Department (ED) patients.

**Objective:**

The present aim is to study the prevalence of stress, types of stressful situations and the relationship with other health issues within the ED population.

**Materials and Methods:**

This is a cross-sectional study performed in an ED at a level-1 urban trauma center for four months. An anonymous survey was offered to adult non critically ill patients who were admitted in the ED. They were divided on the basis of gender, age and level of stress and were asked about their demographics, reasons for their ED visit and health issues including stress, tobacco, alcohol and marijuana use, weight concern and health. Chi-square for the categorical variables and unpaired *t*-tests for continuous variables were conducted.

**Results:**

We interviewed 1797 patients, over 66% reported that they felt stressed on at least a weekly basis, and over 45% indicated that they felt stressed more than twice per week. While both young and old were equally stressed, females reported significantly more stress. Family, finances and work are the most frequently cited stressors. Different age groups reported different types of stressors. Overall, those patients reporting being stressed more frequently reported more high risk behaviors including cigarette and marijuana use and health problems including being overweight, being depressed, and having sleeping problems and chronic fatigue.

**Conclusions:**

Considering the high frequency of ED patients that report frequent stress and high risk behaviors, their ED visit may be an excellent opportunity to provide a referral or an intervention for stress reduction.

## INTRODUCTION

The Emergency Department (ED) is a focal point for community access to care, not only for acute medical emergencies, but also for chronic health problems and sub acute medical conditions. The Society for Academic Emergency Medicine (SAEM) Public Health and Education Task Force in 2000 recognized that for vulnerable populations their recourse for medical care was often through EDs, and that this makes the ED an important venue for offering key preventative services. Many of the key areas identified by the SAEM task force involved behaviors that have been linked to stress.[1,2]

The perception of stress and both the psychological and physiological responses to stress have been associated with an increased risk of health problems.[3,4] Sudden and chronic exposure to life events that are perceived as stressors have been associated with coping strategies that negatively impact on physical and psychological health, including alcohol, tobacco, and marijuana use.[5] Many patients who visit the ED for treatment of an illness or an injury are experiencing stress associated with life events. As such, these ED patients may be experiencing physical and psychological health problems both from the allostatic load associated with exposure to the physiological response to stressors and the health problems that are part of the negative behavioral strategies used to cope with the experience of stress.[6] Although little is known about stress among ED patients, a recent survey demonstrated that from a menu of possible health care information, over 50% of ED patients wanted information on referrals to programs that reduced stress.[7]

Over the past 10 years, brief interventions for emergency medicine patients to address high risk behaviors (e.g., alcohol and marijuana use) have been tested in the ED and frequently have been shown to be successful in reducing substance use and the negative consequences of substance use.[8] During clinical supervisions with counselors who conduct brief interventions focused on patients' misuse of alcohol and other drugs, it was frequently reported that many ED patients stated that stress was a major life problem that they wanted to address; and that many patients related their experience of stress to their use of alcohol and drugs.

However, information on the prevalence of stress and types of stressors experienced by ED patients is lacking. To effectively design an intervention for stress the aims of the present study are to ascertain the prevalence of stress, types of stressful situations and the relationship with other health issues within the ED population, and examine the effects of age and gender on the report of stressful situations and the relationship with other health issues within the ED population.

## MATERIALS AND METHODS

### Study design

This is a cross-sectional study conducted at varied time periods selected from all 10 am to 10 pm shifts and days of the week over a four-month time period.

### Study setting and eligibility criteria

This study was performed at a busy urban level I ED/Trauma Center with over 100,000 patient visits annually. Eligible patients were adult patients not critically ill who were admitted to the ED that were at least 18 years old and if they spoke English and were not under arrest, nor psychiatrically impaired.

### Study protocol

An anonymous health survey, the Wellness Questionnaire, is a non-validated survey instrument that was developed by the research team submitting this report. It was conducted to identify the prevalence of risky lifestyle behaviors, among ED patients, divided on the basis of gender, age and level of stress. It is a 19 item survey asking about patient demographic characteristics, reason for the ED visit and health issues including stress, tobacco, alcohol and marijuana use, weight concern, and health problems. This study was approved by the Hospital Institutional Review Board, and patients were asked to verbally consent to being in this study prior to completing the survey. In preparing this report we adhered to the STROBE statement guidelines for reporting observational studies.[9] The statistical analyses were carried out using the Statistical Analysis System (SAS) version 9.1. Chi-square analyses were used for categorical variables and unpaired *t*-test for continuous variables. Prior to data analysis continuous variables were analyzed for skewness and kurtosis. The collected data were categorized by gender, level of reported stress (more than twice a week vs twice a week or less) and age (using a median split) to allow subgroup analyses to be conducted.

## RESULTS

During the time that research assistants were in the ED screening and recruiting patients there were approximately 2000 patients who met the basic screening criteria of being age 18 years or older, not under arrest or psychiatrically impaired and being seen in the ED for a non-critical illness or injury. Out of these patients approximately 91% completed the Wellness questionnaire, 1% did not speak English, less than 1% were missed by the research assistant (i.e. discharged before being screened), and 7% refused to participate.

There were 1797 patients who completed the survey, 51% females, with a mean age 36.5 years. Most patients were seeking medical care in the ED for an illness (62%) vs. an injury (38%), and 45% of our respondents reported experiencing stress frequently (which we defined as at least twice weekly). These respondents reported a variety of stressful situations (e.g., family 41.7%, finances 41.1%) and health problems (e.g., cigarettes 39.4%, depression 28.3%) [Table 1].

**Table 1:** Demographic and health characteristics of the sample

Gender %	Age	Reason for ED visit %	Stress level %	Stressful situations %	Health problems %
Female = 51	Mean = 36.5 years	III = 62.6	> twice week = 45	Family = 41.7	Cigarettes = 39.4
		Injured = 37.4		Finance = 41.1	Marijuana use = 14.7
				Work = 40.3	Overweight > 30lbs = 16.4
				Relationships = 22.9	Sleep problems = 29.7
				Health = 22.9	Chronic fatigue = 9.4
					Depression = 28.3

We examined differences in demographics, stress and health characteristics of patients who had attended the ED for an injury versus those attending for an illness. These results are detailed in Table 2. Ill patients were more likely to be female (*P* < 0.001), be stressed about work (*P* < 0.03), and their health than injured patients (*P* < 0.01) [Table 2]. Ill patients were also more likely to report most of the measured health problems than injured patients.

**Table 2:** Demographic and health characteristics of ill and injured patients

	III	Injured	Statistics
Gender %	Female = 56	Female = 43	χ^2^ (1) = 29.8, *P* <.001
Age (years)	M = 36.8 (SD = 12.6)	M = 35 (SD = 12.9)	t (1782) =1.51, *P* =.13
Stress level (in %)	> twice a week = 52.7 (95 CI = 49.8 to 55.6)	> twice a week = 50.6 (95 CI = 46.9 to 54.4)	χ^2^ (1) = 0.7, *P* =.39
Stressful situations (in %)	Family = 40.7 (95 CI = 37.8 to 43.6)	Family = 43.6 (95 CI = 39.9 to 47.3)	χ^2^ (1) = 1.3, *P* =.25
	Finance = 41.6 (95 CI = 38.7 to 44.5)	Finance = 40.6 (95 CI = 36.9 to 44.3)	χ^2^ (1) = 0.16, *P*=.69
	Work = 38.2 (95 CI = 35.3 to 41.1)	Work = 43.8 (95 CI = 40.1 to 47.5)	χ^2^ (1) = 4.8, *P* =.03
	Relationships = 23.9 (95 CI = 21.4 to 26.4)	Relationships = 21.6 (95 CI = 18.5 to 24.7)	χ^2^ (1) = 1.08, *P*=.30
	Health = 25.4 (95 CI = 22.8 to 27.9)	Health = 18.7 (95 CI = 15.8 to 21.6)	χ^2^ (1) = 9.3, *P* <.01
Health problems (in %)	Cigarettes = 71.6 (95 CI = 68.9 to 74.2)	Cigarettes = 64.7 (95 CI = 61.1 to 68.3)	χ^2^ (1) = 3.6, *P* =.06
	Marijuana use = 14.8 (95 CI = 12.7 to 16.9)	Marijuana use = 16.3 (95 CI = 13.5 to 19.1)	χ^2^ (1) = 1.10, *P* =.35
	Overweight > 30 lbs = 17.7 (95 CI = 15.5 to 19.9)	Overweight > 30lbs = 14.4 (95 CI = 11.8 to 17)	χ^2^ (1) = 3.5, *P* =.06
	Sleep problems = 34 (95 CI = 31.2 to 36.8)	Sleep problems = 22.8 (95 CI = 19.7 to 25.9)	χ^2^ (1) = 25.2, *P* <.001
	Chronic fatigue = 11.6 (95 CI = 10 to 13.5)	Chronic fatigue = 5.5 (95 CI = 3.8 to 7.2)	χ^2^ (1) = 18, *P* <.001
	Depression = 30.4 (95 CI = 27.2 to 33.1)	Depression = 24.6 (95 CI = 21.4 to 27.8)	χ^2^ (1) = 6.4, *P* =.01

Those respondents who reported that they were stressed more than twice per week in comparison to those who report being stressed less often, reported more often use of cigarettes (*P* < 0.001) and marijuana (*P* = 0.04). In addition, they more often report being overweight by more than 30 lbs (*P* < 0.001) and having sleep problems (*P* < 0.001), chronic fatigue (*P* < 0.001) and being depressed (*P* < 0.001). However, surprisingly, both groups reported similar frequency of alcohol use and weekly alcohol consumption [Table 3].

**Table 3:** Health problems and stress level

Health problems	Stressed > 2 times/wk *N* = 793 %	Stressed < 2 times/wk *N* = 1004 %	Statistic
Cigarette use	46.8 (95 CI = 43.3 to 50.3)	33.3 (95 CI = 30.4 to 36.2)	χ^2^ (1) = 33.4, *P* <.001
Marijuana use	16.7 (95 CI = 14.1 to 19.3)	13.2 (95 CI = 11.1 to 15.3)	χ^2^ (1) = 4; *P* =.04
Overweight > 30 lbs	20.0 (95 CI = 17.8 to 22.8)	13.6 (95 CI = 11.5 to 15.7)	χ^2^ (1) = 13.2, *P* <.001
Sleeping problems	40.3 (95 CI = 36.9 to 43.7)	21.4 (95 CI = 18.9 to 23.9)	χ^2^ (1) = 75.4, *P* <.001
Chronic fatigue	14.2 (95 CI = 11.8 to 16.4)	5.7 (95 CI = 4.3 to 7.1)	χ^2^ (1) = 35.9, P <.001
Depression	43.2 (95 CI = 39.8 to 46.6)	16.5 (95 CI = 14.2 to 18.8)	χ^2^ (1) = 155.1, *P* <.001

When we looked at the sample by gender [Table 4], the females were more likely to be seen in the ED for an illness than males (69 vs. 56%; *P* ≤ 0.001). We also observed significant differences in the frequency of stress reported and the reasons given for being stressed. More females than males report that they feel stressed more than twice per week (51 vs. 39%, *P* < 0.001). Females reported family (*P* < 0.001) and health (*P* = 0.02) as more often stressful than did males. However, males reported work as more often stressful than did females (*P* = 0.02).

**Table 4:** Gender, stress level and reasons for stress

	Males *N* = 878 %	Females *N* = 919 %	Statistic
Reason for ED visit % Ill	56 (95 CI = 53 to 59)	69 (95 CI = 66 to 72)	χ^2^ (1) = 44.1, *P* <0.001
% Stress level > 2 times/wk	39 (95 CI = 36 to 42)	51 (95 CI= 48 to 54)	χ^2^ (1) = 44.1, *P* <0.001
% Reason stressed Health	20 (95 CI = 17 to 24)	25 (95 CI = 22 to 28)	χ^2^ (1) = 5.1, *P* = 0.02
Work	43 (95 CI = 40 to 46)	38 (95 CI = 34 to 42)	χ^2^ (1) = 5.3, *P* =0.02
Relationships	23 (95 CI = 20 to 26)	23 (95 CI = 20 to 26)	χ^2^ (1) = 0.003, *P* =0.96
Family	36 (95 CI = 32 to 40)	47 (95 CI = 42 to 52)	χ^2^ (1) = 17.6, *P* <0.001
Finances	40 (95 CI = 37 to 43)	42 (95 CI = 39 to 45)	χ^2^ (1) = 0.8, *P* =0.38

After using a median split, we compared responses of those 18 to 35 year olds (*N* = 900) with those 36 years and above (*N* = 897) [Table 5]. Overall, the age groups reported equal occurrences of stress (almost 50% of both groups reported being stressed more than twice per week). Respondents in the different age groups report different situations that most often stress them, younger respondents were more likely to be stressed about relationships (26.7 vs. 19.1%; *P* < 0.001) and work than older respondents (45.7 vs. 34.7%; *P* < 0.001). Older respondents were more likely to report stress related to health (26.4 vs. 19.4%, *P* = 0.001).

**Table 5:** Stressors by participant age

Stressors	18-35 yrs % Reporting *N* = 900 %	≥ 36 yrs % Reporting *N* = 894 %	Statistic
Health	19.4 (95 CI = 16.9 to 21.9)	26.4 (95 CI = 23.5 to 29.3)	χ^2^ (1) = 11.2, *P* = 0.001
Work	45.7 (95 CI = 42.4 to 49.0)	34.7 (95 CI = 31.6 to 37.8)	χ^2^ (1) = 20.2, *P* <0.001
Relationships	26.7 (95 CI = 23.8 to 29.6)	19.1 (95 CI = 16.6 to 21.6)	χ^2^ (1) = 137, *P* <0.001
Family	39.6 (95 CI = 36.6 to 42.6)	43.7 (95 CI = 40.4 to 47.0)	χ^2^ (1) = 2.8, *P* =0.09
Finances	41.0 (95 CI = 37.9 to 44.1)	41.1 (95 CI = 38.0 to 44.2)	χ^2^ (1) = 0.01, *P* =0.97

## DISCUSSION

The aims of this study were to report on the prevalence of stress, the factors reported as being causal of stress, and the health problems reported by those experiencing stress among a cross-sectional sample of adult ED patients. We found that almost half of the ED patients that we sampled not only reported experiencing frequent stress but also reported many of the psychosocial causes of stress (such as work, relationship, and finances), health problems associated with stress (such as being overweight and depressed) and unhealthy coping strategies (such as cigarette and marijuana use) associated with experiencing persistent stress. As such, stress needs to be considered as a co-morbid condition in the care of the ED patient. This is consistent, for example, with research that demonstrates that irrespective of participants' usual ability to restrict food intake, under conditions of acute stress, highly- stressed participants were more likely to eat food with high fat and high salt content, and eat more food, than participants who had low situational stress.[10] Other research has demonstrated that acute stress affects cognitive functioning associated with the prefrontal cortex, a brain area associated with several major psychiatric disorders including anxiety and depression.[11]

A substantial body of research has demonstrated the effectiveness of a brief intervention in reducing negative consequences, including injuries, associated with alcohol use among ED patients.[8,12] Initiatives from organizations such as Substance Abuse and Mental Health Services Administration (SAMHSA) have been instrumental in encouraging EDs to include screening for alcohol use problems as part of standard patient care in the ED.[13] This comes from the recognition that alcohol use is strongly related to the reason for the ED visit among a significant proportion of patients,[8] and that failure to at least screen patients for alcohol use problems misses an opportunity to intervene with the patient to prevent future ED visits. Our finding further supports that of other research that ED patients are stressed and may benefit from such an intervention.[7]

In terms of target populations for a stress intervention, our examination of a sample from a busy urban level 1 ED population reveals that both young and old were equally stressed, but females and ill patients reported significantly more stress. For those reporting more frequent stress an intervention should address the etiology of the stress, the medical implications of stress, and techniques to reduce stress. In addition, it is possible that marijuana use or overeating is used to self medicate stress or that stress is contributing to depression. Interventions that address substance use (such as alcohol, marijuana, cigarettes) or depression have to determine if the experience of stress as a response to chronic or acute life experiences are contributing to and sustaining an individual's problem. It is likely that failure to address the issue of stress may reduce the effectiveness of an intervention.

In an effort to minimize the length of the assessment, the present study examines stress in terms of only very brief self reported measure. Future investigations may benefit by utilizing standardized instruments on stress that can provide additional information on the relative experience of stress within an ED population compared to others. These results do not indicate any causal factors for experiencing stress. We do not know, for example, if marijuana use is a coping strategy for dealing with stress, or if imbibing marijuana is a cause of stress. Although anecdotally many of our clinical interventionists reported that ED patients undergoing a brief intervention reported that they were stressed, and associated their alcohol and/or marijuana use with experiencing stress, we do not know if the intervention having reduced alcohol and/or marijuana use increased or decreased patients experience and consequences of stress.

## CONCLUSIONS

Considering the high frequency of ED patients that report frequent stress, health problems and high risk behaviors, their ED visit may be an excellent opportunity to provide a referral or an intervention for stress reduction. There is now a growing body of research that is demonstrating that brief interventions delivered to ED patients can be efficacious in reducing some unhealthy behaviors (e.g., excessive alcohol consumption and alcohol-related negative consequences).[10] Future studies should examine whether a similar approach that focuses on stress would be of benefit.
